# Accounting for Cooperativity in the Thermotropic Volume Phase Transition of Smart Microgels

**DOI:** 10.3390/gels7020042

**Published:** 2021-04-08

**Authors:** Simon Friesen, Yvonne Hannappel, Sergej Kakorin, Thomas Hellweg

**Affiliations:** Department of Chemistry, Physical and Biophysical Chemistry, Bielefeld University, Universitätsstr. 25, 33615 Bielefeld, Germany; simon.friesen@uni-bielefeld.de (S.F.); yvonne.hannappel@uni-bielefeld.de (Y.H.); sergej.kakorin@uni-bielefeld.de (S.K.)

**Keywords:** Flory–Rehner theory, microgel, Hill cooperativity, Flory–Huggins parameter, swelling behavior, NNPAM, NIPAM, NIPMAM

## Abstract

A full quantitative description of the swelling of smart microgels is still problematic in many cases. The original approach of Flory and Huggins for the monomer–solvent interaction parameter χ cannot be applied to some microgels. The reason for this obviously is that the cross-linking enhances the cooperativity of the volume phase transitions, since all meshes of the network are mechanically coupled. This was ignored in previous approaches, arguing with distinct transition temperatures for different meshes to describe the continuous character of the transition of microgels. Here, we adjust the swelling curves of a series of smart microgels using the Flory–Rehner description, where the polymer–solvent interaction parameter χ is modeled by a Hill-like equation for a cooperative thermotropic transition. This leads to a very good description of all measured microgel swelling curves and yields the physically meaningful Hill parameter ν. A linear decrease of ν is found with increasing concentration of the cross-linker *N,N*${}^{\prime }$-methylenebisacrylamide in the microgel particles p(NIPAM), p(NNPAM), and p(NIPMAM). The linearity suggests that the Hill parameter ν corresponds to the number of water molecules per network chain that cooperatively leave the chain at the volume phase transition. Driven by entropy, ν water molecules of the solvate become cooperatively “free” and leave the polymer network.

## 1. Introduction

In recent years, thermoresponsive microgels have received considerable interest in soft matter science due to their enormous application potential [1,2,3,4,5,6,7,8,9]. They can be used, for example, as carriers for enzymes [10,11], as drug delivery systems [12,13], as responsive surface coatings [14], or in catalysis [15,16,17]. The ability to take up and release chemicals at different temperatures results from their temperature-dependent swelling behavior [18,19,20,21,22,23]. The swelling behavior depends on the chosen monomer, and the related lower critical solution temperature (LCST) of the obtained polymer varies for different acrylamides [6,24]. The best-studied systems are certainly *N*-isopropylacrylamide- [25] and vinylcaprolactam-based [26] microgels. These microgels deswell at approximately 33 °C. Alternative monomers are *N-n*-propylacrylamide (LCST ≈ 22 °C) [27] and *N*-isopropylmethacrylamide (LCST ≈ 45 °C) [28]. Microgels based on these monomers show a volume phase transition (VPT) upon heating with a volume phase transition temperature (VPTT) close to the LCST of the respective linear polymer. Above this temperature, the microgels are collapsed. This swelling behavior is described by the Flory–Rehner theory [29,30,31,32,33,34,35]. In some cases, this description is quantitative; in other cases, it becomes quantitative assuming a Matrioshka-like cross-linker gradient [36]. However, recent super-resolution fluorescence microscopy results indicate that the microgels have a rather extended highly cross-linked core which is less heterogeneous than expected, as well as a rather fuzzy shell [37,38]. This is in line with some slightly older studies with neutrons [39,40] which also show a nearly constant network density in about 90% of the particle volume. Moreover, often the behavior can only be predicted qualitatively [19,41,42]. Hence, there is still a need to achieve a better description of the swelling of microgels. The Flory–Huggins theory uses a lattice model for the calculation of the system partition function [29]. It is assumed that every lattice site has the same volume, so that the solvent molecules and the monomer units occupy an equal volume. The interaction between solvent and polymer network is described by the Flory–Huggins interaction parameter χ. For the calculation of χ, the interactions only with the nearest neighbor are considered. Since in some cases the experimental data cannot be rationalized with this approach, several attempts have been made to modify the Flory–Huggins theory [18]. Erman and Flory suspected that higher-order interactions must also be considered, because in the collapsed state of the gels the packing density is very high [43]. Therefore, they proposed to model the interaction parameter by a series expansion with respect to the swelling ratio. However, in this approach in most cases the virial coefficients do not have a physically meaningful interpretation. Such series expansions were successfully used to fit the swelling curves of different microgels (e.g., [19,44,45,46,47]).

The classical Flory–Rehner theory describes thermoresponsive (or thermotropic) volume phase transitions in gels without accounting for the potentially inhomogeneous distribution of the average degree of polymerization $N_{Gel}$ in microgel particles. In thermoresponsive microgels, continuous transitions are observed nearly exclusively, which is attributed to this inhomogeneous distribution of the average degree of polymerization $N_{Gel}$. This observation was until now explained by different transition temperatures for individual meshes, leading to a continuous character of the VPT [20]. However, what has not yet been considered is that the cross-linking leads to a cooperativity of the VPT, since all meshes of the network are mechanically coupled.

Leite et al. [48] proposed a Hill-like model for χ that yields physically comprehensible parameters and takes into account the cooperativity of the VPT. Moreover, this approach does not rely on the degree of heterogeneity of the microgels.

In our present study, the interaction parameter χ of the Flory–Rehner theory is modeled with this Hill-like model, and is used to describe the swelling behavior of p(NNPAM), p(NIPAM), and p(NIPMAM) homopolymer microgels with different cross-linker concentrations.

## 2. Theory

For isotropic swelling, the polymer volume fraction ϕ is related to the hydrodynamic radius $R_{H}$ of the polymer particle as:

(1) $$\frac{\phi }{\phi_{0}}={\left(\frac{R_{H,0}}{R_{H}}\right)}^{3}$$

where $R_{H,0}$ is the hydrodynamic radius of the particle and $\phi_{0}$ is the polymer volume fraction in the collapsed state, respectively. The hydrodynamic radius of the particle $R_{H}$ is given by the Flory–Rehner equation:

(2) $$\ln\left[1-\phi_{0}{\left(\frac{R_{H,0}}{R_{H}}\right)}^{3}\right]+\phi_{0}{\left(\frac{R_{H,0}}{R_{H}}\right)}^{3}+\chi \phi_{0}^{2}{\left(\frac{R_{H,0}}{R_{H}}\right)}^{6}+\frac{\phi_{0}}{N_{Gel}}\left[\frac{R_{H,0}}{R_{H}}-\frac{1}{2}{\left(\frac{R_{H,0}}{R_{H}}\right)}^{3}\right]=0$$

where $N_{Gel}$ is the average degree of polymerization of a polymer chain, or the number of segments between two cross-linking points. The hydrodynamic radius of the particle $R_{H}$ is a free variable, which can be found by solving Equation (2). In 1996, Hino and Prausnitz suggested a modified version of Equation (2) which is more suitable for heterogeneous gels [49]. However, due to the considerations mentioned in the Introduction, we will not use their approach in the present work.

Note that the $N_{Gel}$ and the so-called number of segments per chain $N_{Seg}$ are described by the same relationship $N_{Gel}=N_{Seg}=V_{0}\phi_{0}N_{A}/\left(v_{s}N_{C}\right)$, where $N_{A}$ is the Avogadro’s constant, $v_{s}$ the molar volume of solvent, for water $v_{s}=18\;{\mathrm{cm}}^{3}/\mathrm{mol}$, $V_{0}=\left(4/3\right)\pi R_{H,0}^{3}$ and $N_{C}$ the number of chains in the polymer network [18,30]. Here, we use *N,N’*-methylenebisacrylamide (BIS) as cross-linker. There are two reactive vinyl units for each BIS molecule. Hence, each cross-linker molecule connects pairs of chains [30]. Therefore the number of chains $N_{C}$ in the polymer network is given by $N_{C}=2N_{BIS}$, where $N_{BIS}$ is the number of BIS molecules in the network. The total number of monomers $N_{M}$ (sites of lattice) occupied by the polymer network is given by [18]:

(3) $$N_{M}=\frac{\phi_{0}V_{0}N_{A}}{v_{s}}.$$

If the nominal amount [BIS] has been incorporated, $N_{BIS}$ is given by:

(4) $$N_{BIS}=\frac{N_{M}\left[\mathrm{BIS}\right]}{100\;\mathrm{mol}\%}$$

where [BIS] is the nominal amount of the cross-linker in mol%. With these notations we can apply $N_{Gel}=N_{M}/N_{C}$ and:

(5) $$N_{Gel}=\frac{50\;\mathrm{mol}\%}{\left[\mathrm{BIS}\right]}.$$

**Figure 1:**
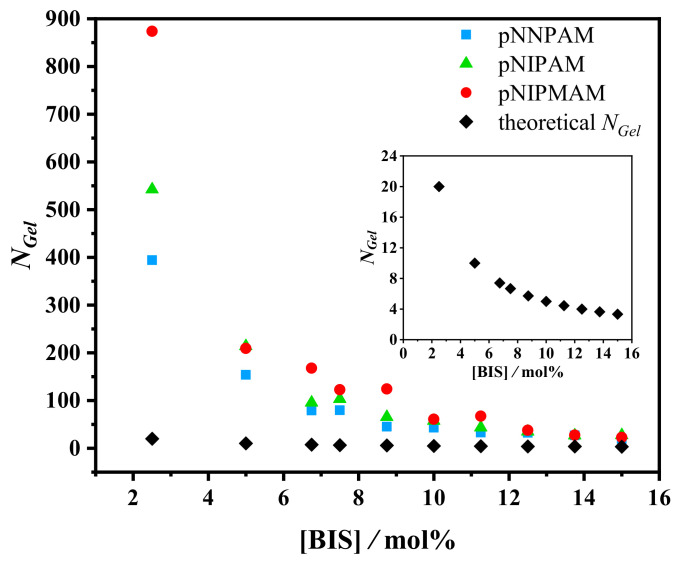
The average degree of polymerization between cross-links $N_{Gel}$ vs. concentration of *N,N’*-methylenebisacrylamide (BIS) in p(NNPAM) (blue), p(NIPAM) (green), and p(NIPMAM) (red) particles. The parameter $N_{Gel}$ was calculated using the Flory–Rehner Equation (2) with the Hill-like Equation (6) for the interaction parameter χ. The theoretical $N_{Gel}$ (black) was calculated with Equation (5). To better see the hyperbolic dependence between $N_{Gel}$ and [BIS], the theoretical $N_{Gel}$ is shown in the inset. More details will be discussed later.

$N_{Gel}$ vs. [BIS] for the different microgel particles is presented in Figure 1. In all cases, $N_{Gel}$ decreases almost hyperbolically with the increasing concentration of BIS.

However, since the BIS cross-linkers in the microgel are not homogeneously distributed [20], Equation (5) describes the average degree of polymerization $N_{Gel}$ only qualitatively, as discussed in the Results and Discussion.

### Hill-Like Equation

It is obvious that the VPT of strongly cross-linked microgels must be cooperative, since the meshes of the network are mechanically coupled. One can imagine that in a network with an inhomogeneous distribution of $N_{Gel}$, a polymer chain first collapses locally at volume phase transition and water molecules leave the chain. Since the collapsed chain is mechanically coupled to the neighboring chains, the VPT is induced in the neighboring chains.

To describe this cooperativity, we use the Hill model for a cooperative aggregation to model the Flory–Huggins interaction parameter χ[50]. The dependence of χ on the temperature *t* is modeled by the Hill-like function for a cooperative thermotropic structure transition with linear baseline:

(6) $$\chi \left(t\right)=\chi_{0}+a\left(t-t_{a}\right)+b\frac{t_{rel}^{\nu }}{t_{rel}^{\nu }+K}$$

where $\chi_{0}$ is the value of the χ parameter at $t=t_{a}$, $t_{a}$ is the first temperature data point, and $t_{e}$ is the last (end) temperature point of the data set, *a* is the slope of the baseline, *b* is the dimensionless amplitude parameter of the Hill transition, *K* is the half-saturation constant, ν is the Hill parameter, and $t_{rel}\left(t\right)$ is the relative temperature given by:

(7) $$t_{rel}\left(t\right)=\frac{t-t_{a}}{t_{e}-t_{a}}.$$

Note that the relative temperature $t_{rel}\left(t\right)$ is used as an analog of polymer concentration. Therefore, the concentration dependence of the original Flory–Huggins parameter is preserved. The Hill parameter ν is the stoichiometric coefficient of the reaction:

(8) $$PS_{\nu }⇄P+\nu S.$$

The symbols *P* and νS denote the states of the polymer and solvent after the VPT, respectively, and the symbol $PS_{\nu }$ denotes the aggregate state at the onset of the VPT. In contrast to a lyotropic transition, for the thermotropic transition the concentration of species is replaced by the relative temperature $t_{rel}$ changing in the region $0\leq t_{rel}\left(t\right)\leq 1$. If χ>0.5, the polymer–solvent binding is no longer energetically favorable. The gel particles collapse at VPT (thermo-shrinking gels). If χ<0.5, the free energy of the binding decreases and the microgels are swelling [18].

## 3. Results and Discussion

**Figure 2:**
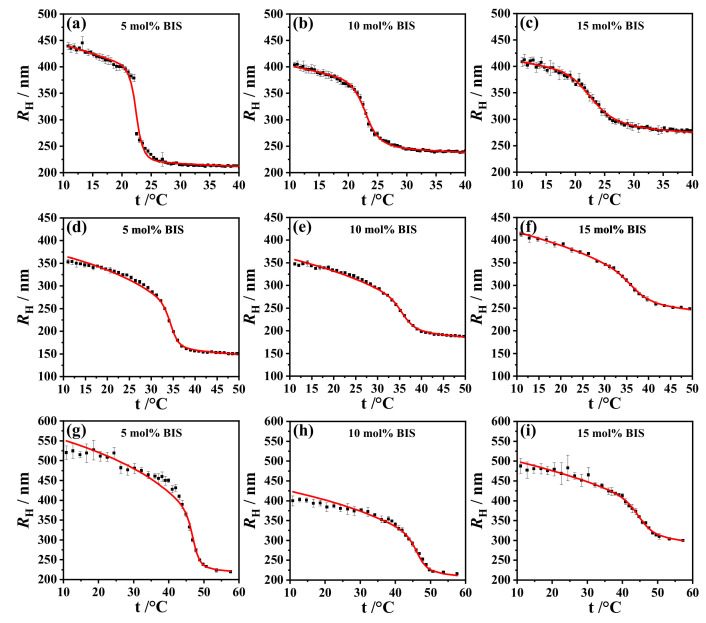
Hydrodynamic radius $R_{H}$ vs. temperature *t* at different concentrations of the cross-linker BIS in p(NNPAM) (**a**–**c**), p(NIPAM) (**d**–**f**), and p(NIPMAM) (**g**–**i**) particles. The fit of the hydrodynamic radii $R_{H}$ (solid curve) was calculated using the Flory–Rehner Equation (2) with the Hill-like Equation (6) for the interaction parameter χ. A nearly perfect fit to the experimental data was achieved for all measured BIS concentrations.

The hydrodynamic radius $R_{H}$ as a function of temperature *t* calculated using the Flory–Rehner Equation (2) with the Hill-like Equation (6) for χ was fitted to the swelling curves at different concentrations of BIS in the range of 2.5 ≤ [BIS]/mol% ≤ 15.0. Here we studied microgel particles of p(NNPAM) (Figures S1 and S2 in the Supplementary Materials), p(NIPAM) (Figures S3 and S4 in the Supplementary Materials), and p(NIPMAM) (Figures S5 and S6 in the Supplementary Materials). The parameters $\phi_{0}$, $N_{Gel}$, *K*, and ν were obtained as fitting parameters; see Tables S1–S3 (also given in the Supplementary Materials). The polymer volume fraction $\phi_{0}$ in the collapsed state was practically independent of the BIS concentration for all microgel types. The average value of $\phi_{0}$ for p(NNPAM) was 0.75 ± 0.02, for p(NIPAM) it was 0.72 ± 0.02, and for p(NIPMAM) it was 0.72 ± 0.03. These values are still controversial since several small-angle neutron scattering (SANS) studies gave values between 0.4 and 0.6 at the particle center [39,51] for $\phi_{0}$. However, for a direct comparison, the p(r) density functions obtained from SANS (or SAXS) need to be integrated over the microgel radius [39]. Moreover, the difference might be due to different weighting of the contribution of the outer fuzzy regions in SANS compared to photon correlation spectroscopy (PCS). In PCS-based studies, a value of $\phi_{0}=0.8$ has been reported [19,35,42,46,52,53,54]. In Equation (6), the parameters *a*, *K*, *b*, and $\chi_{0}$ were also independent of BIS concentration. Parameters *a*, *K*, *b*, and $\chi_{0}$ were fitted for one swelling curve within a series and the values obtained from this first fit and were subsequently used as input parameters and held constant for all other fits within the series. Fitting of the data was performed with the software Mathcad Prime 6.0. As an example, Figure 2 shows a selection of the swelling curves fitted with Equation (2), and Table 1 lists the respective results of the fits for p(NNPAM), p(NIPAM), and p(NIPMAM) microgels with the respective cross-linker concentrations of 5 mol%, 10 mol%, and 15 mol%.

The statistical significances of the fit parameters ν, *K*, and $N_{Gel}$ were estimated using the chi-square functions ${\left(chi\right)}^{2}$, Equation (S.1), and the normalized partial derivatives of the ${\left(chi\right)}^{2}$ (see Figure S7 and explanations in the Supplementary Materials). The three parameters ν, *K*, and $N_{Gel}$ have similarly high sensitivities to small deviations around the minimum. Such sensitivities allow a reliable determination of the fit parameters. In almost all fits, very small values for ${\left(chi\right)}^{2}$ were obtained. For instance, for p(NNPAM) the values of ${\left(chi\right)}^{2}$ were on average 6.0 with 61 data points of the swelling curve, for p(NIPAM) ${\left(chi\right)}^{2}=1.0$ with 41 data points, and for p(NIPMAM) ${\left(chi\right)}^{2}=7.4$ with 32 data points.

Only the $R_{H}\left(t\right)$–swelling curves of p(NNPAM) at [BIS]=2.5 mol% and 5 mol% show a partly discontinuous VPT. These curves could not be satisfactorily described by the Flory–Rehner theory with the Hill-like model for the interaction parameter χ (Figure 2 and S1).

**Figure 3:**
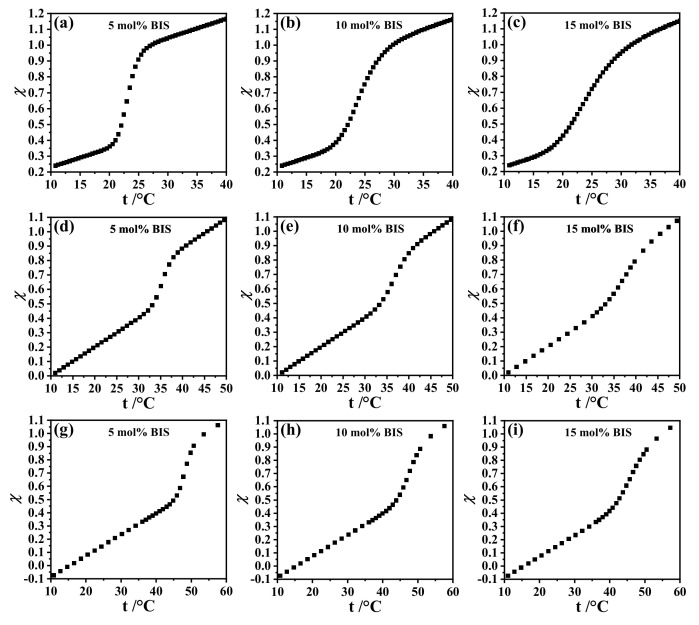
Interaction parameter χ vs. temperature *t* at different concentrations of BIS in p(NNPAM) (**a**–**c**), p(NIPAM) (**d**–**f**), and p(NIPMAM) (**g**–**i**) particles. The interaction parameter χ was calculated with the Hill-like Equation (6).

The swelling curves in all microgel types show that the VPT became increasingly smeared as the cross-linker concentration increased. This behavior is also evident in the curve of the interaction parameter as a function of temperature χ(t) (Figure 3). Wu et al. suggested that this phenomenon can be explained by the inhomogeneous radial distribution of the degree of polymerization $N_{Gel}$ within the gel particles [20]. They assumed that each polymer chain between two cross-linkers has its own volume phase transition temperature. Accordingly, if the distribution of $N_{Gel}$ is very narrow, the VPT is discontinuous. This is the situation for some macroscopic gels [20,55,56,57]. According to Wu et al., larger $N_{Gel}$ have a lower VPTT compared to the VPTT at smaller $N_{Gel}$[20]. Additionally, the microgels showed a sharper VPT at a low cross-linker concentration, suggesting that the distribution of $N_{gel}$ was narrow. With an increase of the cross-linker concentration, $N_{Gel}$ decreased, so, in line with Wu et al., the VPTT should increase [20]. However, the increase of the VPTT was only observed for p(NNPAM) and p(NIPAM) (Table 1). Contrary to p(NNPAM) and p(NIPAM), the VPTT of p(NIPMAM) decreased with the increase of the cross-linker concentration (Table 1).

### 3.1. Average Degree of Polymerization $N_{Gel}$

The values of $N_{Gel}$ obtained from the analysis of swelling curves are systematically larger than given by Equation (5) (Figure 1). If $N_{Gel}$ was radius-independent in the microgel particles, the experimentally obtained values of $N_{Gel}$ could be correctly described by Equation (5). The radial dependence of $N_{Gel}$ in p(NNPAM), p(NIPAM), and p(NIPMAM) particles was also observed by Bergmann et al. and Wrede et al. using super-resolution fluorescence microscopy [37,38].

### 3.2. Half-Saturation Constant *K*

The half-saturation constant is given by:

(9) $$K={\left(\frac{t_{0.5}-t_{a}}{t_{e}-t_{a}}\right)}^{\nu }.$$

Assuming that half of the water molecules have left the gel at the volume phase transition, *K* is then the concentration of $PS_{\nu }$ at the VPTT. Hence, the half-temperature $t_{0.5}$ corresponds to the VPTT. Tables S1–S3 clearly show that the $t_{0.5}$ and VPTT were almost identical in all cases studied here.

### 3.3. Hill Parameter ν

**Figure 4:**
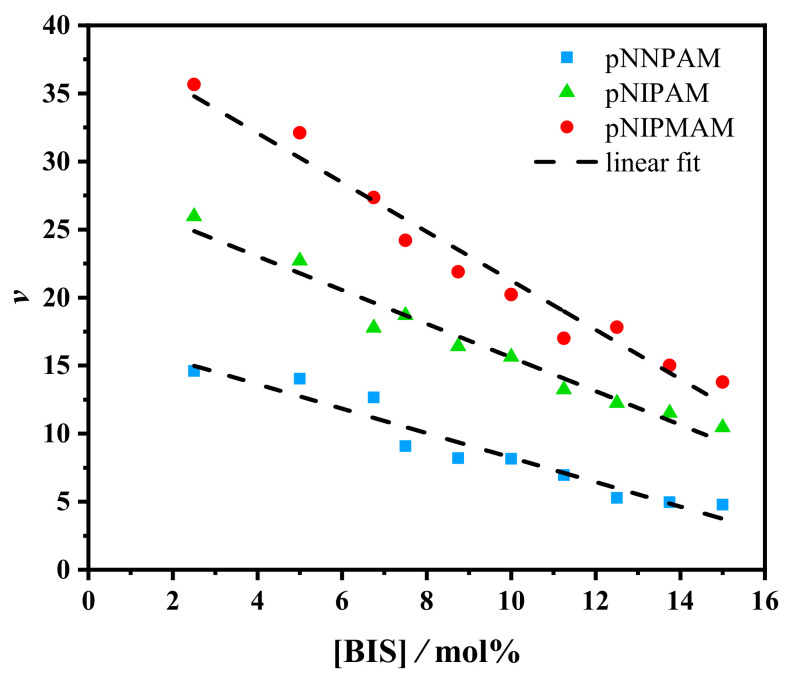
The Hill parameter ν vs. concentration of BIS in p(NNPAM) (blue), p(NIPAM) (green), and p(NIPMAM) (red) particles. The Hill parameter ν has been calculated with the Hill-like Equation (6) for the interaction parameter χ. The dashed lines are guides to the eye.

**Figure 5:**
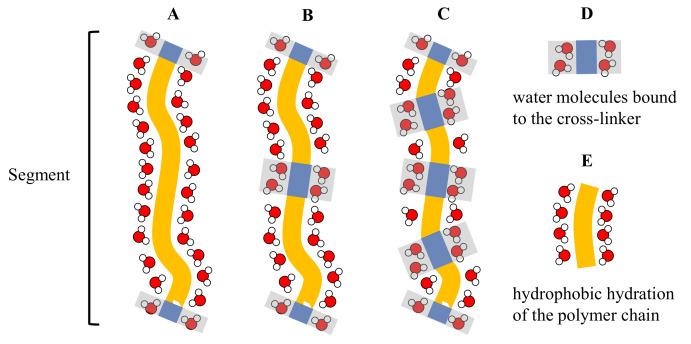
Schematic representation of the water molecules attached to an arbitrary polymer segment. It is assumed that the water molecules bound to the monomer units E leave the segment at the VPT while the water molecules bound to the cross-linker D remain at their position because the cross-linker has no LCST. Segment A contains only one cross-linker. In segment B the concentration of the cross-linker is two times higher and in segment C it is four times higher than in segment A. The number of water molecules that leave the microgel per segment at the VPT should decrease linearly with the increase of the cross-linker concentration.

For all microgels studied in the present work, ν linearly decreased with the increase of [BIS] (Figure 4). The results suggest that ν represents the number of water molecules per segment with a certain length cooperatively leaving the segment at the VPT (Figure 5).

Since the cross-linker molecules have no LCST, we assume that the water molecules bound to the cross-linker do not leave the segment at the VPT. Indeed, Figure 4 suggests that the number of water molecules cooperatively leaving the segment decreased linearly with the increasing cross-linker concentration.

The values of ν in Figure 4 show that p(NIPMAM) bound about 40% more water compared to p(NIPAM) microgel particles. P(NIPAM) bound about 70% more water in relation to the p(NNPAM) segments. Hence, the calculated ν parameter nicely follows the differences in hydrophilicity of the three polymers. This observation is in line with the fact that the p(NNPAM) microgels were more hydrophobic than p(NIPAM) microgels, and p(NIPAM) microgels were more hydrophobic than p(NIPMAM) microgel particles. The hydrophobicity of the gels can be seen from the VPTT. The lower the VPTT, the larger the hydrophobicity of the gels. The relatively small amount of water molecules leaving the microgel per segment at the VPT is at least qualitatively consistent with the results of recent molecular dynamics (MD) simulations [58,59,60].

## 4. Conclusions

We have shown that using the Flory–Rehner theory and the Hill-like model for the interaction parameter χ, the swelling behavior of the p(NNPAM), p(NIPAM), and p(NIPMAM) microgels with different BIS concentrations can be quantitatively described. The Hill-like model for χ provides a deeper insight into the volume phase transition of acrylamide microgels than the use of series expansions for the interaction parameter χ [1]. Interestingly, the original approach of Flory and Huggins for the monomer–solvent interaction does not apply successfully to some microgels [32]. The reason for this is obviously that the cross-linking enhances the cooperativity of the volume phase transitions because all meshes of the network are mechanically coupled. This was ignored in previous descriptions arguing with distinct transition temperatures for individual meshes to describe the broad continuous character of the transition of microgels. Moreover, we observed a linear relationship between the Hill parameter ν and the BIS concentration. This linearity suggests that the Hill parameter ν corresponds to the number of water molecules per network chain that cooperatively leave the chain at the volume phase transition. Additionally, we found that the Hill parameter increased with increasing microgel hydrophilicity. In the future, this approach will be applied to other acrylamide-based microgels, and to copolymer particles as well. Additional improvements might be possible by accounting for the surface charges stemming from the initiator.

## 5. Materials and Methods

### 5.1. Chemicals

*N-n*-propylacrylamide (NNPAM) was synthesized via a Schotten–Baumann reaction published by Hirano et al. [61]. For this reaction, acryloylchloride (Sigma-Aldrich Chemie GmbH, Munich, Germany; purity 98%), *n*-propylamine (Fluka, Sigma-Aldrich Chemie GmbH, Munich, Germany; purity 99%), triethylamine (Grüssing GmbH Analytika, Filsum, Germany; purity 99%), and methylenechloride (p.a.) were used as received. The obtained monomer NNPAM was washed with $NaHCO_{3}$ (10 wt%) and dried over $MgSO_{4}$. After filtration, the solvent was evaporated and the product was distilled in vacuum (115 °C, 10 mbar). *N*-isopropylacrylamide (NIPAM; Sigma-Aldrich Chemie GmbH, Munich, Germany; purity 97%) and *N*-isopropylmethacrylamide (NIPMAM; Sigma-Aldrich Chemie GmbH, Munich, Germany; purity 97%) were purified by recrystallization from hexane. The cross-linker *N,N’*-methylenebisacrylamide (BIS; Sigma-Aldrich Chemie GmbH, Munich, Germany; purity 99%), the initiator ammonium persulfate (APS; Sigma-Aldrich Chemie GmbH, Munich, Germany; purity ≥98%), and pyrene (Sigma-Aldrich Chemie GmbH, Munich, Germany; purity ≥99%) were used without further purification. For all experiments, purified water from an Arium pro VF system (Sartorius AG, Göttingen, Germany) was used.

### 5.2. Synthesis of the Homopolymer Microgels

The homopolymer microgels of NNPAM, NIPAM, and NIPMAM were synthesized via conventional precipitation polymerization without surfactant. All syntheses were performed in a 250 mL three-neck flask equipped with a reflux condenser, mechanical stirrer (210 rpm), and a nitrogen inlet. The monomer (11.05 mmol) and the cross-linker *N,N’*-methylenebisacrylamide (BIS) (2.5 mol%, 5.0 mol%, 6.75 mol%, 7.5 mol%, 8.75 mol%, 10.0 mol%, 11.25 mol%, 12.5 mol%, 13.75 mol%, 15.0 mol% respective to the total monomer amount) were dissolved in 150 mL purified water and heated to 70 °C under continuous stirring and purged with nitrogen. After 1 h the polymerization was initiated by the addition of 2 mL of the 0.2 M solution of APS and left to proceed for 4 h at 70 °C. Subsequently, the solution was cooled to room temperature and stirred overnight. For purification, all samples were treated by four cycles of centrifugation, decantation, and redispersion in purified water using a JA-30.50 Ti Rotor in an Avanti J-30I centrifuge (Beckman Coulter, Brea, CA, USA) at 20,000 rpm and 25 °C.

### 5.3. Photon Correlation Spectroscopy (PCS)

The PCS measurements were performed on a custom-built fixed-angle setup (scattering angle θ: 60°) utilizing a He–Ne Laser (wavelength λ=632.8 nm, 21 mW, Thorlabs, Newton, MA, USA) and two photomultipliers (ALV/SO-SIPD, ALV-GmbH, Langen, Germany) in a pseudo-cross-correlation configuration. The signal was correlated with an ALV-6010 multiple-tau correlator (ALV GmbH, Langen, Germany). Subsequently, the intensity–time correlation functions were converted to the field–time correlation function $g^{1}\left(t\right)$ and analyzed using the CONTIN software [62]. However, an analysis using a second-order cumulant function also leads to the same result within the exp. precision. The temperature was controlled via a thermostat (Phoenix II, Thermo Fisher Scientific, Waltham, MA, USA together with Haake C25P, Thermo Fisher Scientific, Waltham, MA, USA), and the sample was equilibrated for 25 min inside the decaline-filled refractive index matching bath. For each temperature, 5 consecutive measurements were performed. The obtained mean relaxation rates Γ of the $g^{1}\left(t\right)$ functions were converted to the hydrodynamic radius by

(10) $$R_{h}=\frac{k_{B}T}{6\pi \eta \frac{\Gamma }{q^{2}}}\;\;.$$

Here, $k_{B}$ is the Boltzmann constant, η the solvent viscosity (water), *T* the temperature in Kelvin, and $q=\frac{4\pi n}{\lambda }\sin\frac{\theta }{2}$ the magnitude of the scattering vector. *n* is the refractive index of the solvent. 

## Figures and Tables

**Table 1:** Parameters resulting from the fit of swelling curves with the Hill-like Equation (6) and the volume phase transition temperature (VPTT) determined from the inflection point of the $R_{H}\left(t\right)$–swelling curve.

	[BIS]/mol%	$K/10^{-4}$	ν	$N_{\mathrm{Gel}}$	$t_{0.5}{/}^{{}^{\circ}}C$	$\mathrm{VPTT}{/}^{{}^{\circ}}C$
**p(NNPAM)**	5.00	0.057	14.0	153.5	23.2	23.2
	10.0	20.00	8.16	43.28	24.1	23.9
	15.0	260.0	4.77	19.43	24.4	24.0
**p(NIPAM)**	5.00	0.307	22.7	213.8	35.4	35.7
	10.0	20.00	15.6	57.40	36.8	37.1
	15.0	230.0	10.5	27.50	37.8	38.6
**p(NIPMAM)**	5.00	8.000	32.1	209.1	48.3	48.0
	10.0	80.00	20.2	60.67	47.6	47.8
	15.0	220.0	13.8	22.40	46.1	46.4

## Data Availability

Data can be obtained from the authors upon request.

## Supplementary Materials

The following are available online at https://www.mdpi.com/article/10.3390/gels7020042/s1. Figure S1: Hydrodynamic radius $R_{H}$ vs. temperature *t*, measured (points) and calculated (lines), at different concentrations of BIS in p(NNPAM) particles. Figure S2: Interaction parameter χ vs. temperature *t* at different concentrations of BIS in p(NNPAM) particles. Figure S3: Hydrodynamic radius $R_{H}$ vs. temperature *t*, measured (points) and calculated (lines), at different concentrations of BIS in p(NIPAM) particles. Figure S4: Interaction parameter χ vs. temperature *t* at different concentrations of BIS in p(NIPAM) particles. Figure S5: Hydrodynamic radius $R_{H}$ vs. temperature *t*, measured (points) and calculated (lines), at different concentrations of BIS in p(NIPMAM) particles. Figure S6: Interaction parameter χ vs. temperature *t* at different concentrations of BIS in p(NIPMAM) particles. Figure S7: Chi-Square ${\left(chi\right)}^{2}$ vs. the fit-parameters ν, *K* and $N_{Gel}$ of the $R_{H}\left(t\right)$-swelling curve from p(NIPAM) with a concentration of 2.5 mol% BIS. Table S1: Parameters resulting from the fit of the swelling curves of p(NNPAM )with the Hill-like Equation (6). Table S2: Parameters resulting from the fit of the swelling curves of p(NIPAM )with the Hill-like Equation (6). Table S3: Parameters resulting from the fit of the swelling curves of p(NIPMAM )with the Hill-like Equation (6).

## References

[1] Pelton R. (2000). Temperature-sensitive aqueous microgels. Adv. Colloid Interface Sci..

[2] Thorne J.B., Vine G.J., Snowden M.J. (2011). Microgel applications and commercial considerations. Colloid Polym. Sci..

[3] Saunders B.R., Vincent B. (1999). Microgel particles as model colloids: Theory, properties and applications. Adv. Colloid Interface Sci..

[4] Wedel B., Zeiser M., Hellweg T. (2012). Non NIPAM Based Smart Microgels: Systematic Variation of the Volume Phase Transition Temperature by Copolymerization. Z. FÜR Phys. Chem..

[5] Karg M., Pich A., Hellweg T., Hoare T., Lyon L.A., Crassous J.J., Suzuki D., Gumerov R.A., Schneider S., Potemkin I.I. (2019). Nanogels and Microgels: From Model Colloids to Applications, Recent Developments, and Future Trends. Langmuir Acs J. Surfaces Colloids.

[6] Nayak S., Lyon L.A. (2005). Weiche Nanotechnologie mit weichen Nanopartikeln. Angew. Chem..

[7] Plamper F.A., Richtering W. (2017). Functional Microgels and Microgel Systems. Accounts Chem. Res..

[8] Richtering W., Saunders B.R. (2014). Gel architectures and their complexity. Soft Matter.

[9] Pich A., Albrecht K. (2011). Chemical Design of Responsive Microgels.

[10] Park T.G., Hoffman A.S. (1990). Immobilization and characterization of beta-galactosidase in thermally reversible hydrogel beads. J. Biomed. Mater. Res..

[11] Welsch N., Becker A.L., Dzubiella J., Ballauff M. (2012). Core–shell microgels as “smart” carriers for enzymes. Soft Matter.

[12] Langer R. (1990). New methods of drug delivery. Science.

[13] Smeets N.M.B., Hoare T. (2013). Designing responsive microgels for drug delivery applications. J. Polym. Sci. Part Polym. Chem..

[14] Uhlig K., Wegener T., He J., Zeiser M., Bookhold J., Dewald I., Godino N., Jaeger M., Hellweg T., Fery A. (2016). Patterned Thermoresponsive Microgel Coatings for Noninvasive Processing of Adherent Cells. Biomacromolecules.

[15] Lu Y., Mei Y., Drechsler M., Ballauff M. (2006). Thermosensitive core-shell particles as carriers for ag nanoparticles: Modulating the catalytic activity by a phase transition in networks. Angew. Chem..

[16] Sabadasch V., Wiehemeier L., Kottke T., Hellweg T. (2020). Core–shell microgels as thermoresponsive carriers for catalytic palladium nanoparticles. Soft Matter.

[17] Lu Y., Spyra P., Mei Y., Ballauff M., Pich A. (2007). Composite Hydrogels: Robust Carriers for Catalytic Nanoparticles. Macromol. Chem. Phys..

[18] Quesada-Pérez M., Maroto-Centeno J.A., Forcada J., Hidalgo-Alvarez R. (2011). Gel swelling theories: The classical formalism and recent approaches. Soft Matter.

[19] Hertle Y., Zeiser M., Hasenöhrl C., Busch P., Hellweg T. (2010). Responsive P(NIPAM-co-NtBAM) microgels: Flory–Rehner description of the swelling behaviour. Colloid Polym. Sci..

[20] Wu C., Zhou S. (1997). Volume Phase Transition of Swollen Gels: Discontinuous or Continuous?. Macromolecules.

[21] Duracher D., Elassari A., Pichot C. (1999). Preparation of poly(N-isopropylmethacrylamide) latexes kinetic studies and characterization. J. Polym. Sci. Part Polym. Chem..

[22] Shibayama M., Tanaka T. (1998). Small–angle neutron scattering study on weakly charged poly(N–isopropyl acrylamide–co–acrylic acid) copolymer solutions. J. Chem. Phys..

[23] Tanaka T., Fillmore D.J. (1979). Kinetics of swelling of gels. J. Chem. Phys..

[24] Gehrke S.H., Dušek K. (1993). Synthesis, equilibrium swelling, kinetics, permeability and applications of environmentally responsive gels. Responsive Gels: Volume Transitions II.

[25] Pelton R.H., Chibante P. (1986). Preparation of aqueous latices with N-isopropylacrylamide. Colloids Surfaces.

[26] Pich A., Lu Y., Boyko V., Arndt K.F., Adler H.J.P. (2003). Thermo-sensitive poly(N-vinylcaprolactam-co-acetoacetoxyethyl methacrylate) microgels: 2. Incorporation of polypyrrole. Polymer.

[27] Wrede O., Reimann Y., Lülsdorf S., Emmrich D., Schneider K., Schmid A.J., Zauser D., Hannappel Y., Beyer A., Schweins R. (2018). Volume phase transition kinetics of smart N-n-propylacrylamide microgels studied by time-resolved pressure jump small angle neutron scattering. Sci. Rep..

[28] Berndt I., Richtering W. (2003). Doubly Temperature Sensitive Core-Shell Microgels. Macromolecules.

[29] Flory P.J., Rehner J. (1943). Statistical Mechanics of Cross–Linked Polymer Networks I. Rubberlike Elasticity. J. Chem. Phys..

[30] Flory P.J. (1953). Principles of Polymer Chemistry.

[31] Dušek K., Patterson D. (1968). Transition in swollen polymer networks induced by intramolecular condensation. J. Polym. Sci. Part Polym. Phys..

[32] Flory P.J. (1970). Fifteenth Spiers Memorial Lecture. Thermodynamics of polymer solutions. Discuss. Faraday Soc..

[33] Graessley W.W. (2004). Polymeric Liquids and Networks: Structure and Properties.

[34] Dušek K. (1993). Responsive Gels: Volume Transitions II.

[35] Fernández-Barbero A., Fernández-Nieves A., Grillo I., López-Cabarcos E. (2002). Structural modifications in the swelling of inhomogeneous microgels by light and neutron scattering. Phys. Rev. Stat. Nonlinear Soft Matter Phys..

[36] Fernandes P.A.L., Schmidt S., Zeiser M., Fery A., Hellweg T. (2010). Swelling and mechanical properties of polymer gels with cross-linking gradient. Soft Matter.

[37] Bergmann S., Wrede O., Huser T., Hellweg T. (2018). Super-resolution optical microscopy resolves network morphology of smart colloidal microgels. Phys. Chem. Chem. Phys. PCCP.

[38] Wrede O., Bergmann S., Hannappel Y., Hellweg T., Huser T. (2020). Smart microgels investigated by super-resolution fluorescence microscopy: Influence of the monomer structure on the particle morphology. Soft Matter.

[39] Stieger M., Richtering W., Pedersen J.S., Lindner P. (2004). Small-angle neutron scattering study of structural changes in temperature sensitive microgel colloids. J. Chem. Phys..

[40] Arleth L., Xia X., Hjelm R.P., Wu J., Hu Z. (2005). Volume transition and internal structures of small poly(N-isopropylacrylamide) microgels. J. Polym. Sci. Part Polym. Phys..

[41] Neuburger N.A., Eichinger B.E. (1988). Critical experimental test of the Flory-Rehner theory of swelling. Macromolecules.

[42] Lopez C.G., Richtering W. (2017). Does Flory-Rehner theory quantitatively describe the swelling of thermoresponsive microgels?. Soft Matter.

[43] Erman B., Flory P.J. (1986). Critical phenomena and transitions in swollen polymer networks and in linear macromolecules. Macromolecules.

[44] Lietor-Santos J.J., Sierra-Martin B., Vavrin R., Hu Z., Gasser U., Fernandez-Nieves A. (2009). Deswelling Microgel Particles Using Hydrostatic Pressure. Macromolecules.

[45] Voudouris P., Florea D., van der Schoot P., Wyss H.M. (2013). Micromechanics of temperature sensitive microgels: Dip in the Poisson ratio near the LCST. Soft Matter.

[46] Nigro V., Angelini R., Bertoldo M., Bruni F., Ricci M.A., Ruzicka B. (2017). Dynamical behavior of microgels of interpenetrated polymer networks. Soft Matter.

[47] Nigro V., Angelini R., Rosi B., Bertoldo M., Buratti E., Casciardi S., Sennato S., Ruzicka B. (2019). Study of network composition in interpenetrating polymer networks of poly(N isopropylacrylamide) microgels: The role of poly(acrylic acid). J. Colloid Interface Sci..

[48] Leite D.C., Kakorin S., Hertle Y., Hellweg T., da Silveira N.P. (2018). Smart Starch-Poly( N-isopropylacrylamide) Hybrid Microgels: Synthesis, Structure, and Swelling Behavior. Langmuir ACS J. Surfaces Colloids.

[49] Hino T., Prausnitz J.M. (1996). Swelling equilibria for heterogeneous polyacrylamide gels. J. Appl. Polym. Sci..

[50] Hill A.V. (1910). The possible effects of the aggregation of the molecules of haemoglobin on its dissociation curves. J. Physiol..

[51] Cors M., Wiehemeier L., Hertle Y., Feoktystov A., Cousin F., Hellweg T., Oberdisse J. (2018). Determination of Internal Density Profiles of Smart Acrylamide-Based Microgels by Small-Angle Neutron Scattering: A Multishell Reverse Monte Carlo Approach. Langmuir.

[52] López-León T., Fernández-Nieves A. (2007). Macroscopically probing the entropic influence of ions: Deswelling neutral microgels with salt. Phys. Rev. Stat. Nonlinear Soft Matter Phys..

[53] Sierra-Martín B., Romero-Cano M.S., Fernández-Nieves A., Fernández-Barbero A. (2006). Thermal control over the electrophoresis of soft colloidal particles. Langmuir ACS J. Surfaces Colloids.

[54] Witte J., Kyrey T., Lutzki J., Dahl A.M., Houston J., Radulescu A., Pipich V., Stingaciu L., Kühnhammer M., Witt M.U. (2019). A comparison of the network structure and inner dynamics of homogeneously and heterogeneously crosslinked PNIPAM microgels with high crosslinker content. Soft Matter.

[55] Matsuo E.S., Tanaka T. (1988). Kinetics of discontinuous volume–phase transition of gels. J. Chem. Phys..

[56] Shibayama M., Tanaka T., Han C.C. (1992). Small angle neutron scattering study on poly(N-isopropyl acrylamide) gels near their volume-phase transition temperature. J. Chem. Phys..

[57] Hirotsu S. (1993). Coexistence of phases and the nature of the 1st-order phase-transition in poly(N-isopropylacrylamide) gels. Adv. Polym. Sci..

[58] Chiessi E., Paradossi G. (2016). Influence of Tacticity on Hydrophobicity of Poly(N-isopropylacrylamide): A Single Chain Molecular Dynamics Simulation Study. J. Phys. Chem. B.

[59] Tavagnacco L., Zaccarelli E., Chiessi E. (2018). On the molecular origin of the cooperative coil-to-globule transition of poly(N-isopropylacrylamide) in water. Phys. Chem. Chem. Phys. PCCP.

[60] de Oliveira T.E., Marques C.M., Netz P.A. (2018). Molecular dynamics study of the LCST transition in aqueous poly(N-n-propylacrylamide). Phys. Chem. Chem. Phys. PCCP.

[61] Hirano T., Nakamura K., Kamikubo T., Ishii S., Tani K., Mori T., Sato T. (2008). Hydrogen-bond-assisted syndiotactic-specific radical polymerizations ofN-alkylacrylamides: The effect of theN-substituents on the stereospecificities and unusual large hysteresis in the phase-transition behavior of aqueous solution of syndiotactic poly(N-n-propylacrylamide). J. Polym. Sci. Part Polym. Chem..

[62] Provencher S.W. (1982). CONTIN: A general purpose constrained regularization program for inverting noisy linear algebraic and integral equations. Comput. Phys. Commun..

