# Supplementary material for: Accounting for Cooperativity in the Thermotropic Volume Phase Transition of Smart Microgels
Source: Gels. 2021 Apr 8;7(2):42. doi: 10.3390/gels7020042 (PMC8167792; doi:10.3390/gels7020042)
Supplement: Supplementary file 1 [file gels-07-00042-s001.pdf]

Supplementary Materials: Accounting for cooperativity in the thermotropic volume phase transition of smart microgels

Simon Friesen , Yvonne Hannappel , Sergej Kakorin and Thomas Hellweg *

Results of the fitting of $R_{\mathrm{H}}(t)$-swelling curves and the functions $\chi(t)$. The hydrodynamic radius R_{H} has been calculated using the Flory-Rehner Eq. (2) with the Hill-like Eq. (6) for the interaction parameter χ. The fitting has been performed with the software Mathcad Prime 6.0, using the optimization MinErr function. The statictical significances of the fit parameters ν, K and N_{Gel} are discussed.

1. Poly(*N*-*n*-propylacrylamide)

Table S.1. Parameters resulting from the fit of the swelling curves with the Hill-like Eq. (6) and $a = 0.012\ \mathrm{K}^{-1}$, $b = 0.576$, $\phi_0 = 0.75 \pm 0.02$ and $\chi_0 = 0.239$ and the VPTT determined from the inflection point of the $R_{\mathrm{H}}(t)$-swelling curves.

**Sample p(NNPAM) [BIS]/mol%**	$K/10^{-4}$	ν	N_{Gel}	$t_{0.5}/°\mathrm{C}$	VPTT/°C
**1) 2.5**	0.016	14.6	393.9	22.6	22.4
**2) 5.0**	0.057	14.0	153.5	23.2	23.2
**3) 6.75**	0.169	12.7	78.96	23.1	23.0
**4) 7.5**	7.366	9.07	79.60	24.0	23.9
**5) 8.75**	10.00	8.20	45.41	23.5	23.0
**6) 10.0**	20.00	8.16	43.28	24.1	23.9
**7) 11.25**	30.00	6.94	32.64	23.7	23.5
**8) 12.5**	130.0	5.28	32.12	23.7	23.5
**9) 13.75**	190.0	4.96	27.11	24.0	23.7
**10) 15.0**	260.0	4.77	19.43	24.4	24.0

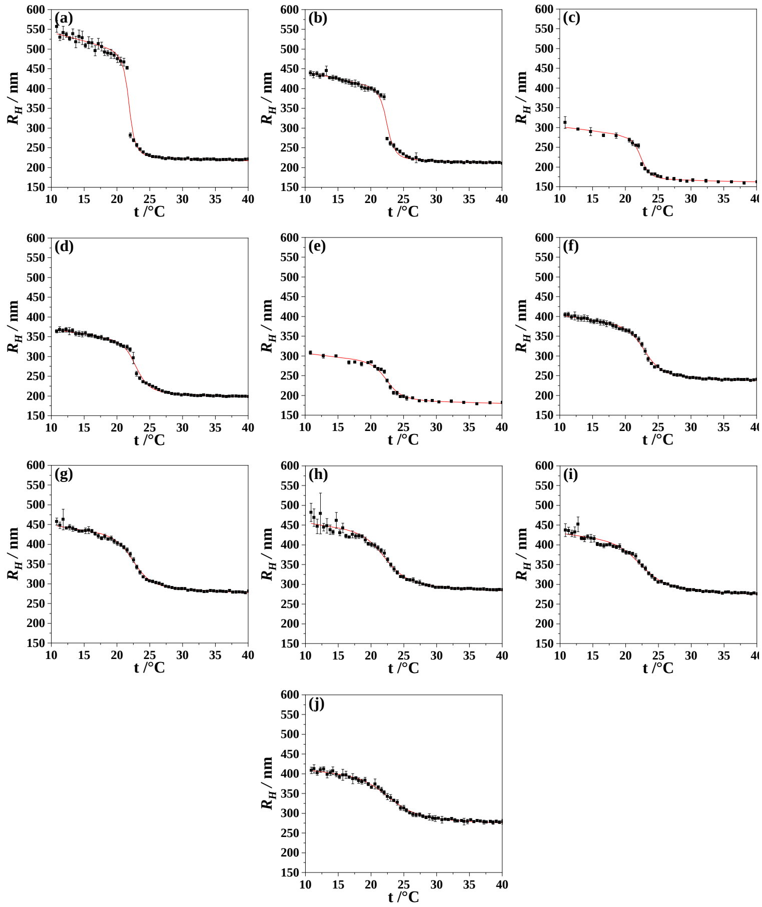

Figure S1. Hydrodynamic radius R_H vs. temperature t, measured (points) and calculated (lines), at different concentrations of BIS in p(NNPAM) particles. The hydrodynamic radius R_H has been calculated using the Flory-Rehner Eq. (2) with the Hill-like Eq. (6) for the interaction parameter χ. A nearly perfect fit of the experimental data is achieved at all BIS-concentrations: [BIS]/mol% = 2.5 (a), 5.0 (b), 6.75 (c), 7.5 (d), 8.75 (e), 10.0 (f), 11.25 (g), 12.5 (h), 13.75 (i), 15.0 (j).

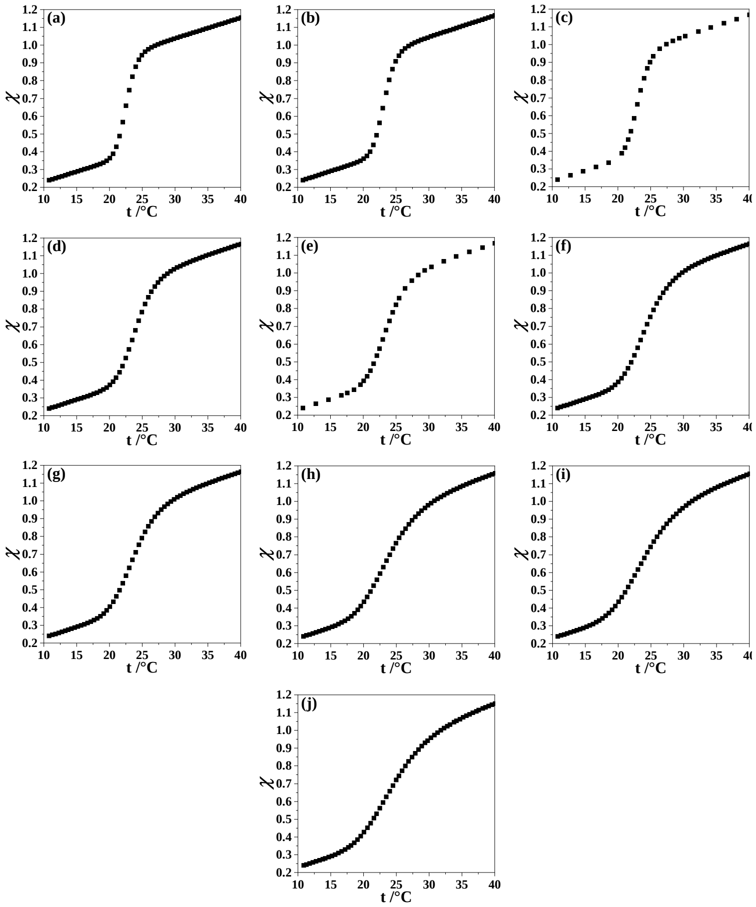

Figure S2. Interaction parameter χ vs. temperature t at different concentrations of BIS in p(NNPAM) particles; [BIS]/mol% = 2.5 (a), 5.0 (b), 6.75 (c), 7.5 (d), 8.75 (e), 10.0 (f), 11.25 (g), 12.5 (h), 13.75 (i), 15.0 (j). The interaction parameter χ has been calculated with the Hill-like Eq. (6).

2. Poly(*N*-isopropylacrylamide)

Table S.2. Parameters resulting from the fit of the swelling curves with the Hill-like Eq. (6) and $a = 0.02\,\mathrm{K}^{-1}$, $b = 0.290$, $\phi_0 = 0.72 \pm 0.02$ and $\chi_0 = 0.02$ and the VPTT determined from the inflection point of the $R_\mathrm{H}(t)$-swelling curve.

**Sample p(NIPAM) [BIS]/mol%**	$K/10^{-4}$	ν	N_{Gel}	$t_{0.5}/°C$	VPTT/°C
**1) 2.500**	0.014	26.0	542.7	33.8	34.1
**2) 5.000**	0.507	22.7	213.8	35.4	35.7
**3) 6.750**	1.771	17.8	95.89	34.6	35.2
**4) 7.500**	3.413	18.7	103.1	36.2	36.4
**5) 8.750**	8.019	16.4	65.80	35.9	38.0
**6) 10.00**	20.00	15.6	57.43	36.8	37.1
**7) 11.25**	40.00	13.2	43.46	36.5	36.8
**8) 12.50**	50.00	12.2	35.11	36.1	36.9
**9) 13.75**	150.0	11.5	26.67	37.7	36.3
**10) 15.00**	230.0	10.5	27.53	37.8	38.6

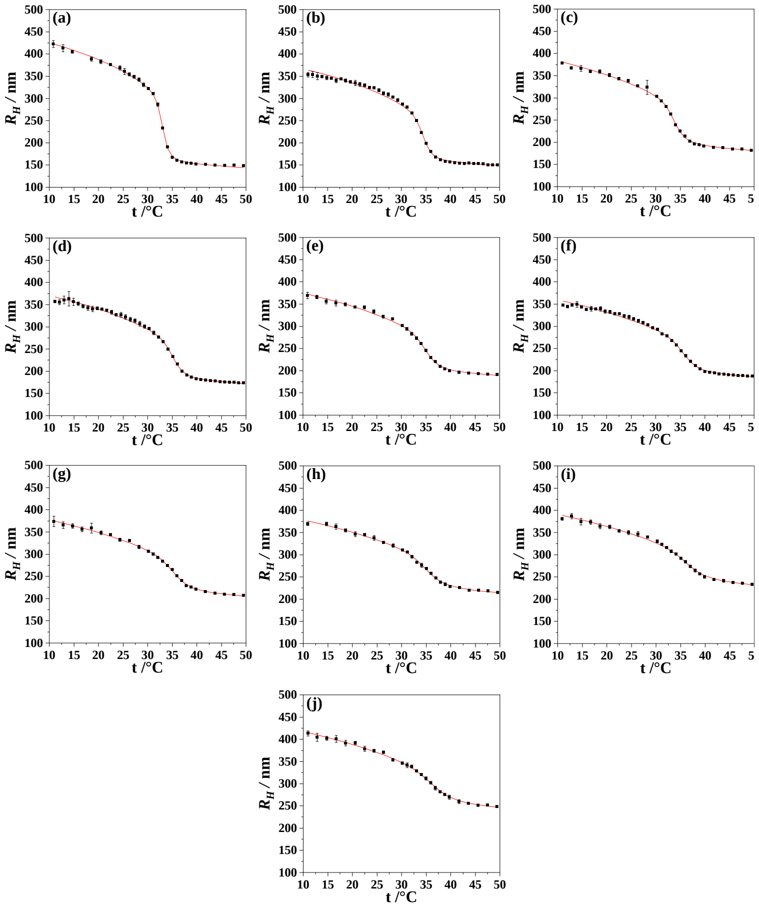

Figure S3. Hydrodynamic radius R_H vs. temperature t, measured (points) and calculated (lines), at different concentrations of BIS in p(NIPAM) particles. The hydrodynamic radius R_H has been calculated using the Flory-Rehner Eq. (2) with the Hill-like Eq. (6) for the interaction parameter χ. A nearly perfect fit of the experimental data is achieved at all BIS-concentrations: [BIS]/mol% = 2.5 (a), 5.0 (b), 6.75 (c), 7.5 (d), 8.75 (e), 10.0 (f), 11.25 (g), 12.5 (h), 13.75 (i), 15.0 (j).

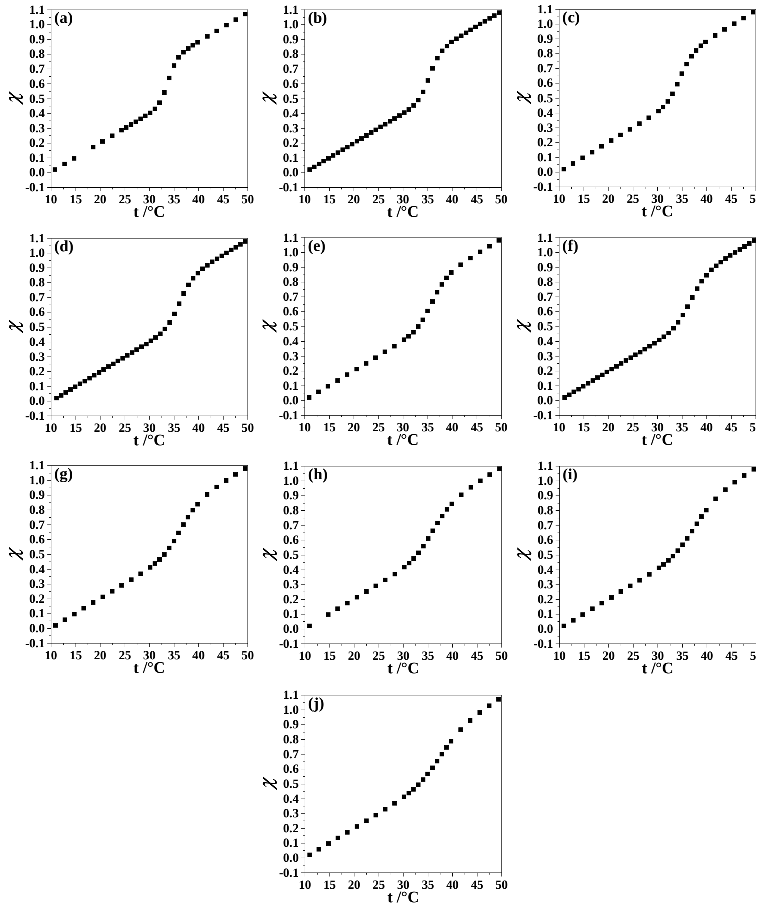

Figure S4. Interaction parameter χ vs. temperature t at different concentrations of BIS in p(NIPAM) particles; [BIS]/mol% = 2.5 (a), 5.0 (b), 6.75 (c), 7.5 (d), 8.75 (e), 10.0 (f), 11.25 (g), 12.5 (h), 13.75 (i), 15.0 (j). The interaction parameter χ has been calculated with the Hill-like Eq. (6).

3. Poly(*N*-isopropylmethacrylamide)

Table S.3. Parameters resulting from the fit of the swelling curves with the Hill-like Eq. (6) and $a = 0.016\ \mathrm{K}^{-1}$, $b = 0.387$, $\phi_0 = 0.72 \pm 0.03$ and $\chi_0 = -0.074$ and the VPTT determined from the inflection point of the $R_\mathrm{H}(t)$-swelling curve.

Sample p(NIPMAM) [BIS]/mol%	$K/10^{-3}$	ν	N_{Gel}	$t_{0.5}$/°C	VPTT/°C
**1) 2.500**	0.23	35.6	873.6	47.8	47.6
**2) 5.000**	0.80	32.1	209.1	48.3	48.0
**3) 6.750**	2.00	27.4	167.9	48.0	47.9
**4) 7.500**	5.00	24.2	122.7	48.5	47.9
**5) 8.750**	6.00	21.9	124.0	47.9	47.9
**6) 10.00**	8.00	20.2	60.66	47.6	47.8
**7) 11.25**	9.00	17.0	67.00	46.2	46.9
**8) 12.50**	8.00	17.8	37.66	46.5	46.7
**9) 13.75**	14.0	15.0	27.66	46.0	46.6
**10) 15.00**	22.0	13.8	22.40	46.1	46.4

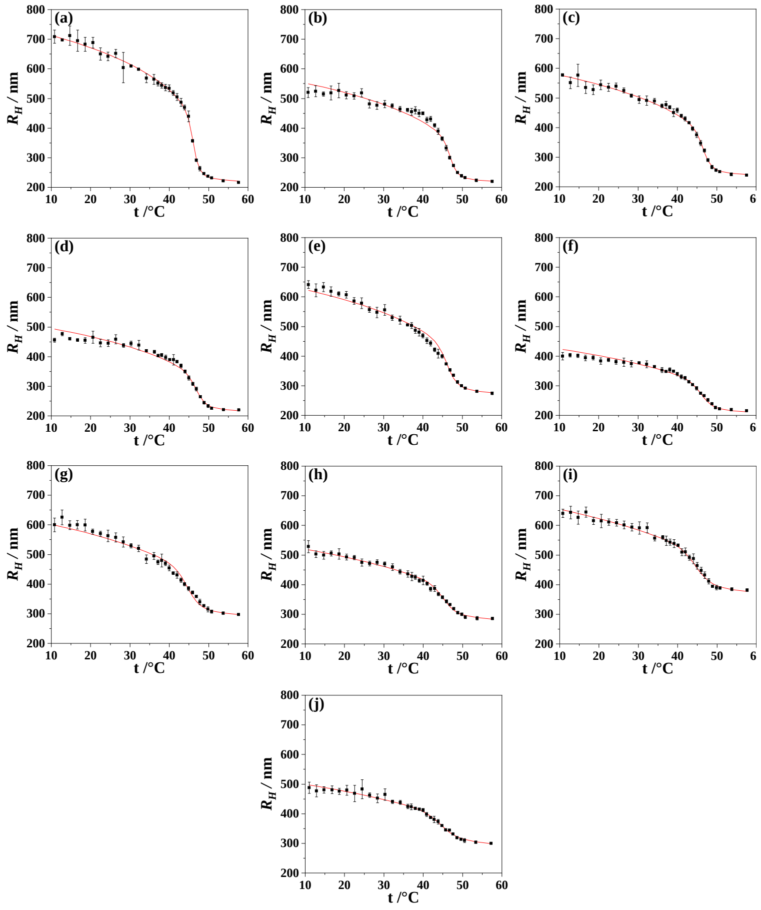

Figure S5. Hydrodynamic radius R_H vs. temperature t, measured (points) and calculated (lines), at different concentrations of BIS in p(NIPMAM) particles. The hydrodynamic radius R_H has been calculated using the Flory-Rehner Eq. (2) with the Hill-like Eq. (6) for the interaction parameter χ. A nearly perfect fit of the experimental data is achieved at all BIS-concentrations: [BIS]/mol% = 2.5 (a), 5.0 (b), 6.75 (c), 7.5 (d), 8.75 (e), 10.0 (f), 11.25 (g), 12.5 (h), 13.75 (i), 15.0 (j).

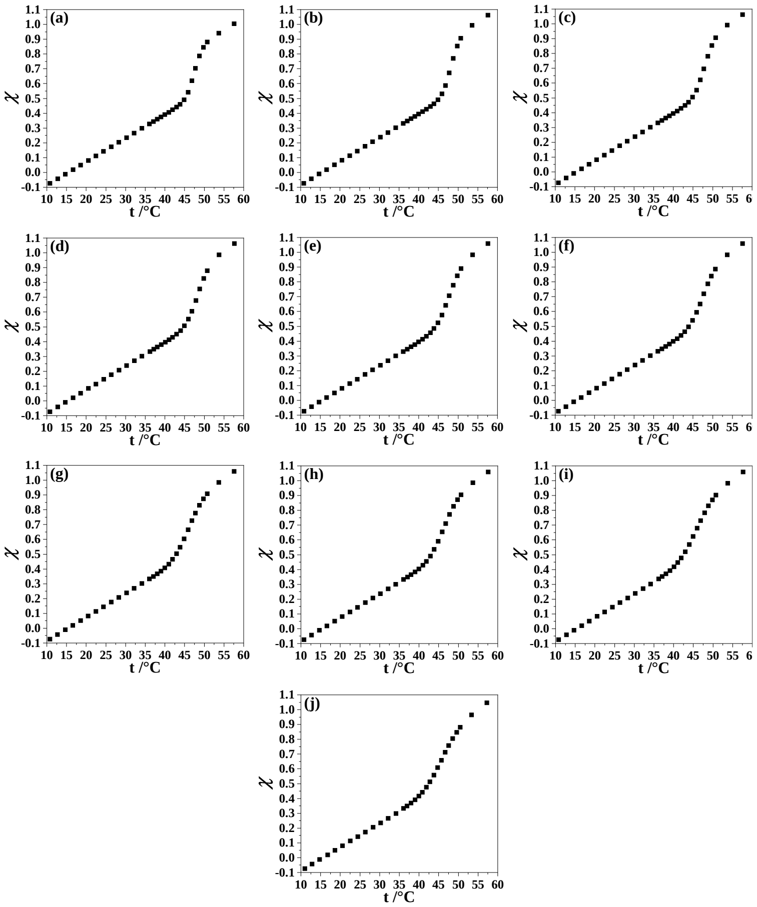

Figure S6. Interaction parameter χ vs. temperature t at different concentrations of BIS in p(NIPMAM) particles; [BIS]/mol% = 2.5 (a), 5.0 (b), 6.75 (c), 7.5 (d), 8.75 (e), 10.0 (f), 11.25 (g), 12.5 (h), 13.75 (i), 15.0 (j). The interaction parameter χ has been calculated with the Hill-like Eq. (6).

4. Significance of the fitting parameters

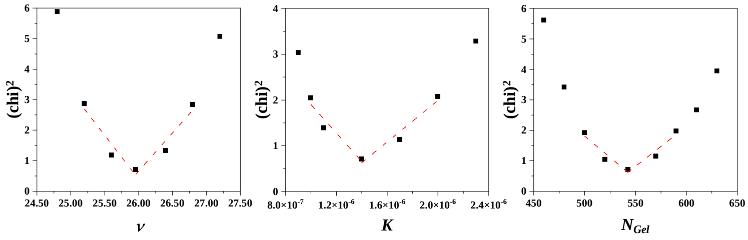

Figure S7. Chi-Square $(chi)^2$ vs. the fit-parameters ν, K and N_{Gel} of the $R_{\text{H}}(t)$-swelling curve from p(NIPAM) with a concentration of 2.5 mol% BIS. The values of the fitting parameters are $\nu_{min} = 26.0$, $K_{min} = 1.4 \times 10^{-6}$ and $N_{Gel,min} = 542.7$. The dashed lines are the slopes of the first 3 points from the minimum. All fitting parameters are in the minimum of chi-square $(chi)^2$.

Chi-square $(chi)^2$ was calculated by the following equation.

$$(chi)^2 = \sum_{t_a}^{t_e} \frac{\left(R_{\text{H},exp}(t) - R_{\text{H},fit}(t, \nu, K, N_{Gel})\right)^2}{R_{\text{H},fit}(t, \nu, K, N_{Gel})} \quad \text{(S.1)}$$

Where $R_{\mathrm{H},exp}(t)$ is the experimentally determined curve and $R_{\mathrm{H},fit}(t,\nu,K,N_{Gel})$ is the fitted curve. Sensitivities of ν, K and N_{Gel} to small deviations of their values around the minimum of $(chi)^2$ have been estimated using the normalized slopes:

$$\nu_{min}\left(\frac{\partial(chi)^2}{\partial\nu}\right)_{K,N_{Gel}} = 7.0\times10^1 \tag{S.2}$$

$$K_{min}\left(\frac{\partial(chi)^2}{\partial K}\right)_{\nu,N_{Gel}} = 3.7 \tag{S.3}$$

$$N_{Gel,min}\left(\frac{\partial(chi)^2}{\partial N_{Gel}}\right)_{\nu,K} = 1.5\times10^1 \tag{S.4}$$

The absolute values of the partial derivatives of $(chi)^2$ have been calculated with the first 3 points from the minimum; see Fig. S7. Normalizations $\partial\nu/\partial\nu_{min}$, $\partial K/\partial K_{min}$ and $\partial N_{Gel}/\partial N_{Gel,min}$ were needed in order to enable a comparison of the sensitivities of the three fitting parameters without an additional scaling. The largest sensitivity, 7.0×10^1, has the Hill-parameter ν; compare Eqs. (S.2), (S.3) and (S.4).
